# Shep RNA-Binding Capacity Is Required for Antagonism of *gypsy* Chromatin Insulator Activity

**DOI:** 10.1534/g3.118.200923

**Published:** 2019-01-10

**Authors:** Dahong Chen, Margarita Brovkina, Leah H. Matzat, Elissa P. Lei

**Affiliations:** Nuclear Organization and Gene Expression Section, Laboratory of Cellular and Developmental Biology, National Institute of Diabetes and Digestive and Kidney Diseases, National Institutes of Health, Bethesda, Maryland, 20892

**Keywords:** Chromatin insulator, Shep, RNA-binding, Enhancer blocking, Barrier activity

## Abstract

Chromatin insulators are DNA-protein complexes that regulate chromatin structure and gene expression in a wide range of organisms. These complexes also harbor enhancer blocking and barrier activities. Increasing evidence suggests that RNA molecules are integral components of insulator complexes. However, how these RNA molecules are involved in insulator function remains unclear. The *Drosophila* RNA-binding protein Shep associates with the *gypsy* insulator complex and inhibits insulator activities. By mutating key residues in the RRM domains, we generated a Shep mutant protein incapable of RNA-binding, and this mutant lost the ability to inhibit barrier activity. In addition, we found that one of many wildtype Shep isoforms but not RRM mutant Shep was sufficient to repress enhancer blocking activities. Finally, wildtype Shep rescued synthetic lethality of *shep*, *mod(mdg4)* double-mutants and developmental defects of *shep* mutant neurons, whereas mutant Shep failed to do so. These results indicate that the RNA-binding ability of Shep is essential for its ability to antagonize insulator activities and promote neuronal maturation. Our findings suggest that regulation of insulator function by RNA-binding proteins relies on RNA-mediated interactions.

Chromatin insulators are DNA-protein complexes that play critical roles in shaping three-dimensional genome organization. Insulators are well known to block enhancer-promoter interactions when placed between the two elements (Bell *et al.* 1999). Furthermore, insulators can also function as barriers that prevent spreading of transcriptionally repressive chromatin to allow gene expression (West *et al.* 2002). There is increasing evidence indicating that RNA molecules are important functional components of insulator complexes in both *Drosophila* and mammals (Matzat *et al.* 2013; Kung *et al.* 2015). Various RNA-binding proteins have been shown to regulate insulator activities (Lei and Corces 2006; Moshkovich *et al.* 2011; Matzat *et al.* 2012; King *et al.* 2014), but a precise role for RNA-binding has not yet been elucidated.

We previously identified the RNA-binding protein Shep as a physical interactor of the *gypsy* insulator. Shep contains two RNA recognition motifs (RRMs) and binds thousands of transcripts (Dale *et al.* 2014; Olesnicky *et al.* 2018) as well as the core *gypsy* insulator protein components Su(Hw), Mod(mdg4)67.2, and CP190 (Matzat *et al.* 2012). Shep associates with the chromatin of thousands of genomic loci and overlaps extensively with core insulator proteins (Matzat *et al.* 2012; Dale *et al.* 2014). Furthermore, Shep regulates transcription of many of the genes with which Shep and insulator proteins associate (Chen *et al.* 2017a). Therefore, Shep may mediate a functional interaction between the *gypsy* insulator complex and RNA.

Shep was shown to act as an antagonist of *gypsy* insulator activity. Loss of Shep leads to increased enhancer blocking and barrier activities (Matzat *et al.* 2012). In addition, loss of Shep leads to synthetic lethality in the Mod(mdg4)67.2 null background, suggesting a critical functional interaction between the two factors to regulate development (Matzat *et al.* 2012). The *shep* gene encodes a large number of isoforms, and it remains unclear which isoform(s) participate in insulator antagonism. Previous work showed that ectopic expression of the Shep E isoform in a wildtype background can antagonize barrier activity (Matzat *et al.* 2012). However, existing *shep* mutants disrupt multiple isoforms, and specific isoform rescue experiments have not yet been performed.

In this study, we mutated key residues in the RRMs of Shep isoform E and verified loss of RNA-binding capability. We ectopically expressed wildtype and a Shep E^RRM^ mutant isoform *in vivo* and found that the RNA-binding mutant lost the ability to disrupt barrier activity. Furthermore, we determined that expression of the wildtype Shep E but not the RRM mutant is capable of rescuing the defect in enhancer blocking antagonism of *shep* mutants. Finally, wildtype Shep E expression but not expression of the RRM mutant isoform rescues synthetic lethality of *shep*, *mod(mdg4)* double mutants and developmental defects of *shep* mutant neurons. Our results suggest that the RNA-binding capability of Shep is necessary to antagonize *gypsy* insulator activities and promote neuronal maturation.

## Materials and Methods

### Drosophila strains

Stocks were raised at 25° on standard cornmeal medium. pUASt-attB *shep* E and pUASt-attB *shep* E RRM mutant constructs were transformed using PhiC31 integrase into the attP40 docking site on chromosome 2L (BestGene, Inc.) and verified by genomic PCR and Western Blotting upon driving with Gal4. The *ct^6^* phenotype was scored in flies on the first day after eclosion. Other fly strains used in this study include luciferase transgenes (insulated and uninsulated *UAS-luciferase*) (Markstein *et al.* 2008), *shep^d05714^* (FBal0186064), *Ser*::*Gal4* (BSC 6791), *Mef2*::*Gal4* (BSC 27390), *arm*::*Gal4* (BSC 1560), UAS-Su(Hw)-RNAi (10724 GD), *shep^BG00836^* (FBal0157046), and *ct^6^*; *mod(mdg4)^u1^* (BSC 59960).

### Cloning

RNA-binding mutations in the Shep E isoform (Y64A, F66A, F69A in the RNP1 domain of RRM1 plus V162A, F164A, R166A in the RNP1 domain of RRM2) were designed based on structural and RNA-binding studies of the *Drosophila* Elav and Sxl RNA-binding proteins (Lee *et al.* 1997; Crowder *et al.* 1999; Lisbin *et al.* 2000). The Shep E RRM mutant was generated by PCR-based site directed mutagenesis of a pENTR/D-TOPO clone (Matzat *et al.* 2012) and cloned into pUASt-attB (Bischof *et al.* 2007) for expression in flies and pET101 for production of recombinant N-terminal tagged His-Shep E RRM mutant protein. All plasmids were sequenced for verification.

### Recombinant protein production

Both wildtype and RRM mutant His-tagged Shep E were produced in *E. coli BL21(DE3)* (Rosetta, Novagen) cells and purified with Ni-NTA-agarose (Qiagen) using manufacturer protocols.

### EMSA

Single stranded RNA probes were *in vitro* transcribed using ^32^P-UTP incorporation with T7 polymerase (MegaScript, Ambion) followed by removal of unincorporated nucleotides (NucAway, Ambion). 10 pmol of labeled probe was mixed with 10 μg tRNA in binding buffer (10% glycerol, 20 mM NaCl, 60 mM KCl, 20 mM HEPES pH 7.5) and heated to 95° for 2 min, then rapidly cooled on ice. 47 pmol protein was added and incubated on ice for 30 min. Samples were run at 4° initially at 1000 V for 1.5 min then at 250 V for approximately 3 h on a pre-run 6% GTG acrylamide gel (90 mM Tris, 30 mM taurine, 0.5 mM EDTA).

### Immunostaining

P14 pupae were dissected and fixed with 4% paraformaldehyde for 1 h and incubated with anti-BURS (Peabody *et al.* 2008) (1:5,000) overnight. Secondary antibodies (Invitrogen) were used at 1:1,000, and samples were imaged as maximum-intensity z-series projections with a Zeiss 780 confocal microscope.

### Primers for in vitro transcription of EMSA probes

*brm* forward primer: TAATACGACTCACTATAGGGAGACTCCGACGACGAGGAGATTG
*brm* reverse primer: GGGAGAAATAAATGGTGTGTGCG
*CG10555* forward primer: TAATACGACTCACTATAGGGAGAGAAGCAAGTAGCAGGCGAGAAAATG
*CG10555* reverse primer: GTTTTCAAAGGGGAGCGGAACC

### Data and reagent availability

The authors state that all data necessary for confirming the conclusions presented in the article are represented fully within the figures. All fly strains and reagents are available upon request.

## Results

### Generation of Shep RNA-binding mutant

**Figure 1:**
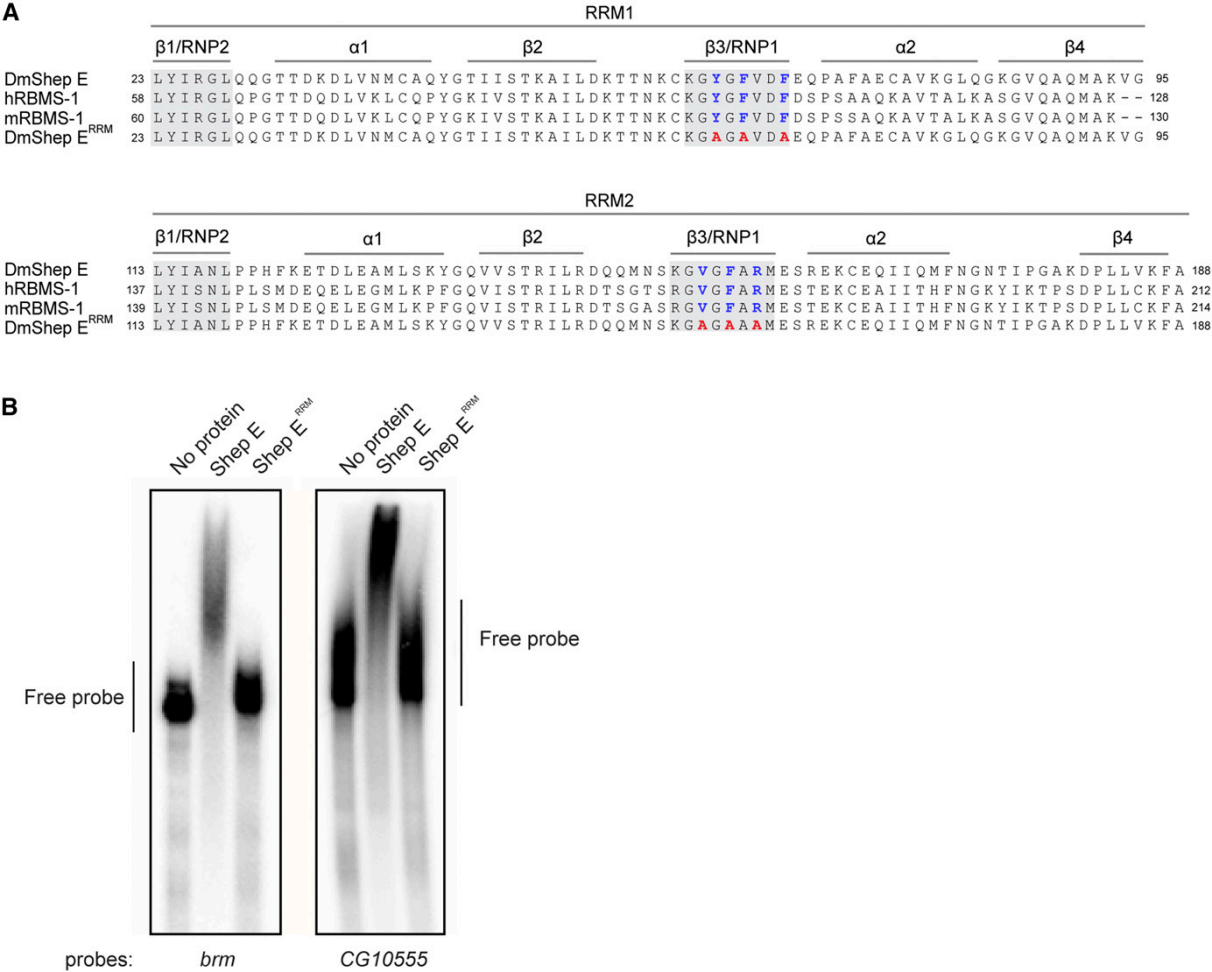
Shep E^RRM^ mutant is defective for RNA binding *in vitro*. A. Conservation of Shep E RRM domains compared to human and mouse orthologs. Consensus resides of RNP1 and RNP2 in RRM domains are shaded for Shep human and mouse orthologs. Residues mutated to alanine of the RNP1 domains are indicated as blue mutated to red. B. EMSA of wildtype Shep E *vs.* Shep E^RRM^ mutant. 455nt ssRNA fragment of *brm* or 470nt ssRNA fragment of *CG10555* was mixed with buffer, recombinant wildtype Shep E, or Shep E^RRM^ mutant and run on a 6% GTG polyacrylamide gel.

In order to test whether the RNA-binding capability of Shep is required for antagonism of *gypsy* insulator function, we generated point mutations in both conserved RNA-binding domains. We selected the E isoform of Shep because ectopic expression in muscle tissue was previously demonstrated to antagonize *gypsy* insulator barrier activity (Matzat *et al.* 2012). Shep harbors two RRMs, each with characteristic βαββαβ structure including RNP2 and RNP1 folds at β1 and β3, respectively (Figure 1A). Because the RNP folds are known to form the hydrophobic surface for RNA interaction, we generated three point mutations in each of the RNP1 folds in both RRM1 and RRM2 domains (Y64A, F66A, F69A in the RNP1 domain of RRM1 plus V162A, F164A, R166A in the RNP1 domain of RRM2). Mutation of each of the residues to alanine was chosen to reduce the likelihood of altered protein folding.

In order to verify that the Shep E^RRM^ mutant is disrupted for RNA-binding, we expressed recombinant His-tagged versions of wildtype and mutant Shep in *E. coli* and tested their RNA binding capability in electrophoretic mobility shift assays (EMSA). We generated single stranded ^32^P labeled RNA probes corresponding to fragments of two transcripts, *brahma* (455 nt) and *CG10555* (470 nt), which are full length transcripts shown to stably associate with Shep in nuclear extracts of CNS-derived BG3 cells (Dale *et al.* 2014). Both RNA fragments contain preferred consensus binding sequences for Shep E previously identified by RNAcompete (Ray *et al.* 2013). As expected, we observed retarded mobility of both probes when wildtype Shep but not RRM mutant was added (Figure 1B).

### Shep RNA-binding is required to antagonize barrier activity

**Figure 2:**
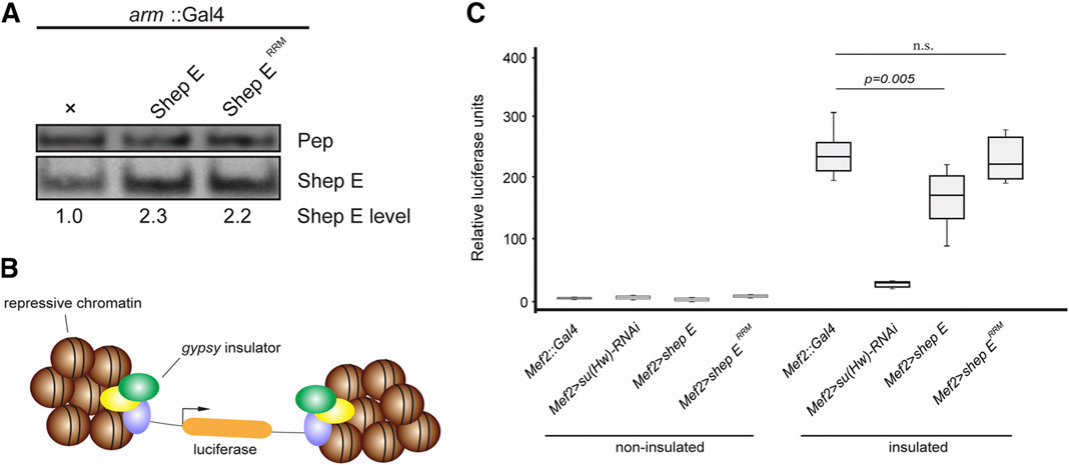
Ectopic expression of wildtype Shep E but not Shep E^RRM^ mutant decreased *gypsy* barrier activity in muscle. A. Anterior thirds of wildtype larvae expressing no transgene, *UAS-shep E*, or *UAS-shep E^RRM^* driven by *arm*::*Gal4* were used for western blotting for Shep E and a loading control Protein on ecdysone puffs (Pep). Normalized band intensity of Shep E relative to Pep was quantified by CCD imaging of chemiluminescence followed by Photoshop analysis. B. Schematic diagram of *UAS-luciferase* system shows flanking *gypsy* insulator sequences act as a barrier to allow *luciferase* expression. C. Relative luciferase activity of non-insulated (left) or insulated (right) reporters in individual larvae expressing *UAS-su(Hw) RNAi*, *UAS-shep E*, or *UAS-shep E^RRM^* driven by *Mef2*::*Gal4*. Luciferase activities are reported as boxplots with boxes representing the first and third quartiles. Luciferase activities across genotypes were compared by One-way ANOVA followed by Tukey HSD *post hoc* tests, with statistical threshold at 0.05. For each genotype, luciferase signals were read for 12 individuals, each normalized to input protein level. Samples showing signal variation more than 100-fold from median of all replicates were discarded as outliers. At least 10 valid replicate samples of each genotype were used for statistics.

We next generated transgenes of either wildtype Shep or RRM mutant under UAS control in order to achieve tissue-specific expression in flies. These constructs were integrated into the *attP40* docking site on chromosome 2L using PhiC31 integrase. We verified by Western blotting that both transgenes express at equivalent levels when driven with *arm*::*Gal4* (Figure 2A).

It was previously shown that ectopic expression of Shep E can antagonize *gypsy* insulator barrier activity in muscle tissue; therefore, we tested whether the RRM mutant retains this activity. We used the muscle-specific *Mef2*::*Gal4* driver to activate expression of a *UAS-luciferase* reporter flanked by *gypsy* insulator sites inserted at the *attP3* docking site (Matzat *et al.* 2012) (Figure 2B). High expression is achieved only if the reporter is insulated, and luciferase expression was highly reduced when Su(Hw) was depleted by RNAi (Figure 2C). When wildtype Shep E was ectopically expressed in this tissue, luciferase expression of the insulated reporter was significantly decreased, indicating reduced insulator activity. However, expression of Shep E^RRM^ had no effect on luciferase activity. These results suggest that RNA-binding of Shep E is required to antagonize *gypsy* insulator barrier activity.

### Shep RNA-binding is required to antagonize enhancer blocking activity and rescue synthetic lethality with loss of Mod(mdg4)67.2

**Figure 3:**
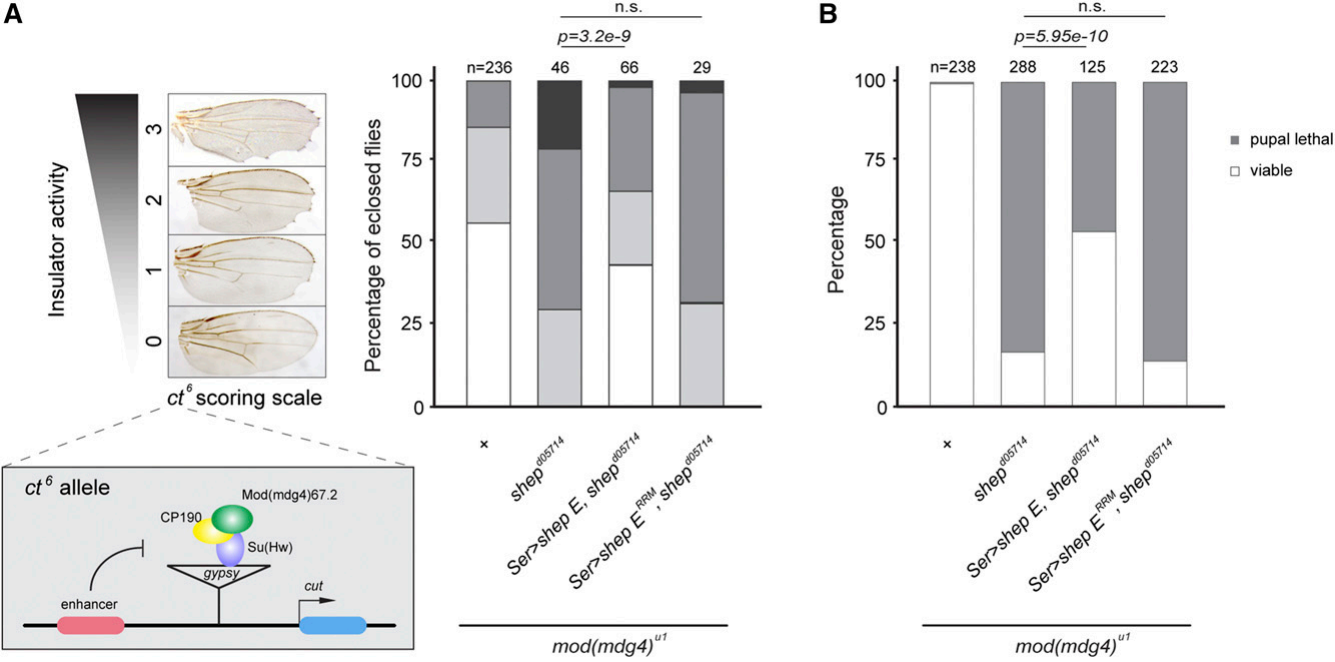
Wildtype Shep E but not Shep E^RRM^ mutant rescues enhancer blocking antagonism of *shep* mutant and its synthetic lethality with *mod(mdg4)^u1^*. A. Schematic diagram and scoring of *ct^6^* reporter activities. A *gypsy* retrotransposon situated between the *ct* promoter and enhancer disrupts *ct* expression and wing development. Insulator activity for *ct^6^* was scored in female wings of indicated genotypes on a scale of 0-3. “0”, no notching; “1”, slight notching in distal tip of wing; “2”, moderate notching throughout distal proportion of wing; “3”, extensive notching in both distal and proximal wing. N represents the total number of flies scored. *UAS-shep E* or *UAS-shep E^RRM^* was driven with *Ser*::*Gal4* driver. Kruskal-Wallis Test was performed (χ^2^_df = 3_ = 166.61, *p*-value < 2.2e-16) followed by *post hoc* pairwise Wilcox-Mann-Whitney *U*-tests. Statistical significance cutoff of Bonferroni-corrected *p* value was 0.05. B. Viability was scored in female flies of indicated genotypes. N represents the examined sample size for each genotype. *UAS-shep E* or *UAS-shep E^RRM^* was driven with *Ser*::*Gal4* driver in homozygous *mod(mdg4)^u1^* background. Pearson’s Chi Squared Test was performed (χ^2^_df = 3_ = 469.52, *p*-value < 2.2e-16) followed by *post hoc* pairwise Fisher’s Exact Tests. Statistical significance cutoff of Bonferroni-corrected *p* value was 0.05.

Furthermore, we examined whether RNA-binding ability is required for Shep antagonism of insulator enhancer blocking activity. It was previously shown that the *shep^d05714^* loss-of-function mutation restores enhancer blocking activity of the *mod(mdg4)^u1^* null mutant at the *ct^6^* locus (Matzat *et al.* 2012). This allele harbors an insertion of the *gypsy* retrotransposon between the wing margin enhancer and promoter of *ct*, resulting in reduced expression of *ct* and disruption of the wing margin (Figure 3A). Enhancer blocking activity of the *mod(mdg4)^u1^* null mutant is low, but additional mutation of *shep^d05714^* results in improvement of enhancer blocking activity. We found that expression of wildtype *UAS-shep E* in the *shep^d05714^*, *mod(mdg4)^u1^* mutant background using the *Ser*::*Gal4* driver substantially decreased enhancer blocking activity, represented as reduced wing notching (Figure 3A). However, similar expression of the *UAS-shep E^RRM^* mutant did not result in prominent rescue of wing morphology. Moreover, we found that expression of wildtype *UAS-shep E* rescued synthetic lethality observed in the *shep^d05714^, mod(mdg4)^u1^* double mutant (Matzat *et al.* 2012), but expression of the RRM mutant had no effect on viability (Figure 3B).

### Shep regulation of neuronal development requires its RNA-binding capacity

**Figure 4:**
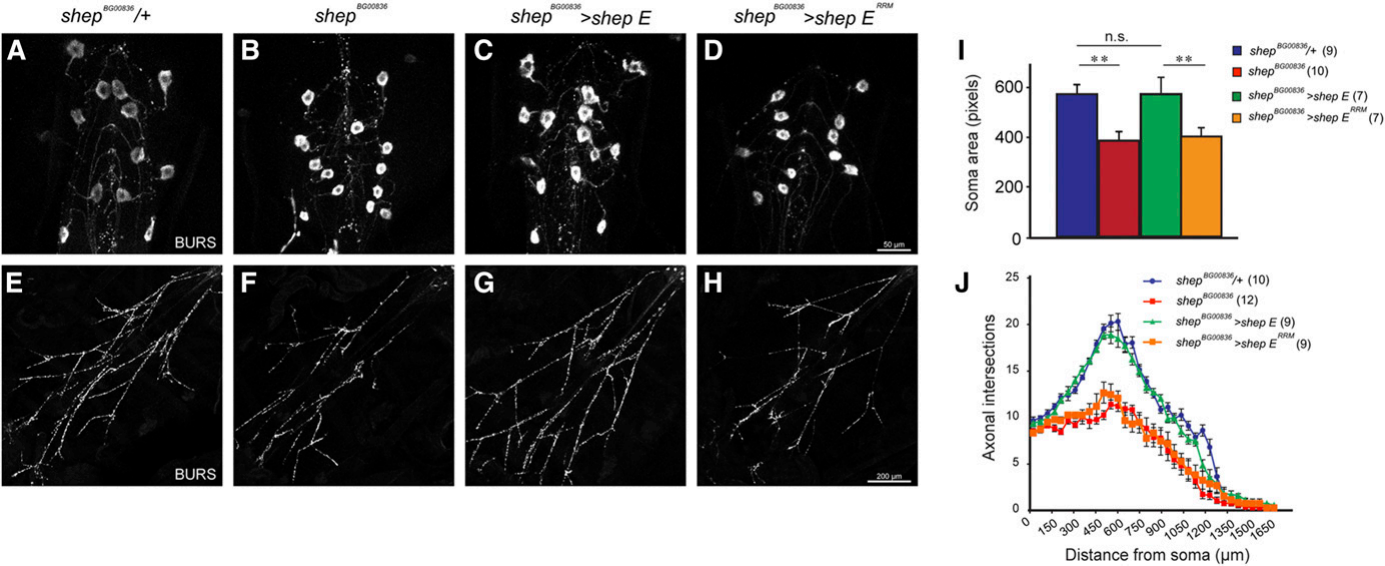
Wildtype Shep E but not Shep E^RRM^ mutant rescues developmental defects of *shep* mutant bursicon neurons. A-H. Homozygous but not heterozygous loss of *shep* (*shep^BG00836^*) leads to smaller cell bodies and fewer projections. Expression of wildtype Shep but not the RRM mutant using a Gal4 driver inserted into the *shep* locus rescues these morphological defects. Bursicon neurons and their projections are visualized by immunostaining with anti-BURS. I. Soma sizes are quantified by Photoshop and compared by One-way ANOVA followed by Tukey HSD *post hoc* tests, with statistical threshold at 0.05, ** *P* < 0.01. Sample sizes are indicated in parentheses. J. Projections of bursicon neurons are quantified by Sholl analysis. Sample sizes are indicated in parentheses.

Since Shep is required for proper neuronal maturation, we examined whether the RRM mutant was sufficient to rescue developmental defects of *shep* mutant neurons. Consistent with previous studies (Chen *et al.* 2014; Chen *et al.* 2017b), we observed smaller cell bodies and fewer projections of homozygous *shep^BG00836^* bursicon neurons that are rescued by expression of wildtype Shep E (Figure 4A-C, E-G, and I-J). However, the RRM mutant Shep failed to rescue these morphological defects (Figure 4D and H-J). These results suggest that Shep RNA-binding capacity is essential for its regulation of neuronal development.

## Discussion

In this manuscript, we tested whether the RNA-binding capacity of Shep is essential for antagonism of *gypsy* insulator activities. We generated six point-mutations in the RNP1 domains of Shep E that caused Shep to become incapable of RNA binding *in vitro*. Subsequent *in vivo* insulator activity assays indicated that expression of wildtype Shep E was sufficient to inhibit *gypsy* enhancer blocking activity in a *shep* mutant genetic background. However, RRM mutant Shep failed to inhibit either enhancer blocking or barrier activities of the *gypsy* insulator. Moreover, RRM mutant Shep did not resuce the synthetic lethality of *shep*, *mod(mdg4)* double mutants or developmental defects of *shep* mutant neurons as did wildtype Shep E. Our results suggest that Shep antagonism of the *gypsy* insulator requires its RNA-binding capacity.

### Shep E expression is sufficient to rescue phenotypes of a *shep* loss-of-function mutant

We showed that expression of Shep E was sufficient to restore the inhibition of *gypsy* enhancer blocking activities and the viability of the *shep*, *mod(mdg4)* double mutant. The observation further extends previous findings that Shep E is sufficient to inhibit the *gypsy* barrier activities (Matzat *et al.* 2012) and are consistent with the finding that expression of Shep E can rescue developmental defects of *shep* mutants (Chen *et al.* 2014). One of the shortest isoforms, Shep E contains both RRM domains, which are present in all isoforms, and therefore may represent a minimal isoform that may be partially functionally redundant with others. Since expression and function of Shep isoforms vary across tissues (Matzat *et al.* 2012), the diverse N termini may contain residues that are needed for tissue-specific functions. To date, FlyBase has reported eight different protein isoforms of Shep. However, we have cloned cDNAs that contain various alternatively spliced microexons that appear to further increase the complexity of protein isoforms and possibly their function (data not shown). Finally, extension of the 3′ UTR of *shep* has been shown to be regulated in a tissue and stage-specific manner (Hilgers *et al.* 2011; Smibert *et al.* 2012), suggesting an additional layer of isoform diversity. Future work will be required to address the function and regulation of these various Shep isoforms.

### Shep may be recruited by RNA molecules to inhibit insulators

Our findings are consistent with a recently proposed model that Shep is co-transcriptionally recruited to regulate insulator activities. Previous studies observed that stably associated RNA immunoprecipitation targets of Shep are often derived from genes to which Shep binds in chromatin, suggesting Shep recruitment *in cis* (Dale *et al.* 2014; Chen *et al.* 2017a). Moreover, Shep-dependent genes revealed by RNA-seq of *shep* mutants are highly enriched as genomic and transcript binding targets of Shep, implying co-transcriptional regulation of gene expression by Shep (Chen *et al.* 2017a). Notably, a significant proportion of these genomic and transcript targets of Shep are also associated with Su(Hw) and Mod(mdg4)67.2 (Chen *et al.* 2017a), and Shep shares many other genomic and transcript targets with Su(Hw) (Matzat *et al.* 2012; Dale *et al.* 2014). Our findings further support that the ability of Shep to antagonize the *gypsy* insulator and regulate neuronal maturation relies on its RNA-binding capacity, raising the possibility that RNA-mediated interactions underlie these functions. These RNA molecules may be co-transcriptionally captured as insulator components that nucleate complexes or further recruit other regulatory factors. Future studies will address these intriguing possibilities.
